# MicroRNAs as Potential Biomarkers in Pituitary Adenomas

**DOI:** 10.3390/ncrna7030055

**Published:** 2021-09-03

**Authors:** Simone Donati, Cinzia Aurilia, Gaia Palmini, Francesca Miglietta, Irene Falsetti, Teresa Iantomasi, Maria Luisa Brandi

**Affiliations:** 1 Department of Experimental and Clinical Biomedical Sciences, University of Florence, 50139 Florence, Italy; simone.donati@unifi.it (S.D.); cinzia.aurilia@unifi.it (C.A.); gaia.palmini@unifi.it (G.P.); francesca.miglietta@unifi.it (F.M.); irene.falsetti@unifi.it (I.F.); teresa.iantomasi@unifi.it (T.I.); 2 Fondazione Italiana Ricerca sulle Malattie dell’Osso (FIRMO Onlus), 50141 Florence, Italy

**Keywords:** pituitary adenomas, biomarker, miRNAs, circulating miRNAs

## Abstract

Pituitary adenomas (PAs) are one of the most common lesions of intracranial neoplasms, occurring in approximately 15% of the general population. They are typically benign, although some adenomas show aggressive behavior, exhibiting rapid growth, drug resistance, and invasion of surrounding tissues. Despite ongoing improvements in diagnostic and therapeutic strategies, late first diagnosis is common, and patients with PAs are prone to relapse. Therefore, earlier diagnosis and prevention of recurrence are of importance to improve patient care. MicroRNAs (miRNAs) are short non-coding single stranded RNAs that regulate gene expression at the post-transcriptional level. An increasing number of studies indicate that a deregulation of their expression patterns is related with pituitary tumorigenesis, suggesting that these small molecules could play a critical role in contributing to tumorigenesis and the onset of these tumors by acting either as oncosuppressors or as oncogenes, depending on the biological context. This paper provides an overview of miRNAs involved in PA tumorigenesis, which might serve as novel potential diagnostic and prognostic non-invasive biomarkers, and for the future development of miRNA-based therapeutic strategies for PAs.

## 1. Introduction

The pituitary gland is one of the main glands of the endocrine system. It is located within the sella turcica of the sphenoid bone, at the base of the skull [1]. This gland is formed by two lobes [2]. The posterior lobe (neurohypophysis) is a projection of hypothalamus and constitutes the nervous part of the pituitary gland, while the anterior lobe (adenohypophysis) is larger than the rear one and is formed cytologically by five different endocrine cell types [2]. Each lobe is responsible for the production of the corresponding hormones. Neurohypophysis produces vasopressin and oxytocin hormones, while adenohypophysis is responsible for the release of the so-called trophic hormones: prolactin (PRL), growth hormone (GH), adrenocorticotrophin (ACTH), thyroid-stimulating hormone (TSH), gonadotrophin (follicle-stimulating hormone (FSH)), and luteinizing hormone (LH). The role of these hormones is to influence metabolism, growth, and reproduction, making the pituitary a highly complex gland to study [3]. Pituitary adenomas (PAs) are one of the most common intracranial tumors occurring in the general population, with a prevalence rate of about 15% [4]. Such lesions are usually benign neoplastic formations, whose symptomatology often manifests as visual disturbances, due to the proximity of the gland to the optic chiasm, headache, given by the compression of surrounding tissues, and systemic endocrine disorders, due to hormone hypersecretion [5,6]. Although more rarely, malignant pituitary tumors have been also described, which are capable of invading the sella turcica and surrounding tissues [6]. These neoplasms are principally classified as functional or non-functional, based on their ability to secrete or not secrete hormones, and in accordance with the type of hormone that is secreted. They are also categorized, depending on their size, into microadenoma (<10 mm), macroadenoma (≥10 mm), and giant adenoma (≥40 mm) [1].

Although two-thirds of PAs can secrete an excessive amount of hormones, not all pituitary adenoma patients develop symptoms because some of these tumors remain small and do not exhibit abnormal hormone levels [7]. Generally, symptoms may appear over the course of many years, thus leading to a delay in diagnosis and therefore of treatment [8]. Due to the increasing sensitivity and usage of magnetic resonance imaging (MRI) and computed tomography (CT), PAs are detected in approximately 10% of asymptomatic patients during imaging exams as part of screening of an unrelated condition [9]. Therefore, significant efforts are still needed to identify relevant biomarkers for improving the diagnosis, and thus for prompting an early treatment for PAs.

The conventional treatment of PAs consists in their resection by transsphenoidal surgery, except for prolactinomas, which involve a therapy based on dopamine agonists, such as cabergoline and bromocriptine [1,7]. It is also possible to use irradiation therapy, which is, however, reserved for patients with PAs that cannot be controlled by using surgical approach or drugs [10,11]. Generally, PAs originate from a single cell, although polyclonality can also be observed [12,13]. However, the pituitary adenoma tumorigenesis remains unclear [14,15]. Emerging evidence highlights that miRNAs could be involved in the pathogenesis of PAs. In 2005, Bottoni et al. [16] were the first to demonstrate that a new class of noncoding RNAs were expressed in normal pituitary, including miRNAs. miRNAs are small endogenous non-coding RNAs capable of negatively regulating gene expression through their direct binding to the 3′ untranslated region (3′-UTR) of the target messenger RNA (mRNA), which is then degraded or translationally inhibited through the complete or incomplete complementarity, with subsequent lack of protein production [17,18,19,20]. Furthermore, miRNAs could bind with other regions, including 5′ untranslated region (5′-UTR), gene promoters, and coding sequence [18]. Recent studies have reported that several miRNAs are involved in the onset and invasiveness of pituitary neoplasms, suggesting that these molecules could act both as oncosuppressors and as oncogenes [2,6].

In recent years, increasing attention has been focused on identifying distinctive expression patterns of miRNAs in human biological fluids in order to use these molecules as novel diagnostic and prognostic biomarkers for various pathological conditions, including PAs [5,21].

Since the symptoms of PAs may appear many years after their development, a non-invasive specific biomarker could be helpful in diagnosis, prognosis, and monitoring of patient response to the treatment. This would be important, especially for tumors with more aggressive behavior and NFPAs, where hormonal tests have limitations.

In 2008, four published studies reported the existence of circulating miRNAs (c-miRNAs) in blood [22,23,24,25]. In recent years, miRNAs have also been detected in many other biological fluids, including saliva [26], cerebrospinal fluid [27], ovarian follicular fluid [28], breast milk [29], urine, tears, bronchial lavage, and seminal fluid [30]. In fact, contrary to cellular RNAs, c-miRNAs are very stable in human body fluids, RNase-rich environments, due to their binding with specific proteins (i.e., Argonaute (Ago 2) protein or lipoproteins, nucleophosmin 1 (NPM1), and high-density lipoproteins (HDLs)) [31,32,33], or their packaging into extracellular vesicles, including exosomes, microvesicles, and apoptotic bodies [26,34].

They have also been demonstrated to be resistant in deleterious conditions, such as acidic or alkaline pH, high temperature, and multiple freeze-thaw cycles [22,23]. As a result of these characteristics, and the fact that their expression levels are strictly linked to pathology [35], c-miRNAs are ideal candidates as new non-invasive blood-based biomarkers for different types of diseases.

Given the important adverse effects brought by traditional therapeutic approaches, the aim of this review is to provide an overview of miRNAs involved in the tumorigenesis of PAs over the last three years (2018–2021) that could be considered as possible biomarkers, as well as candidate targets for the future development of novel therapeutic strategies against this pathological condition.

According to different types of PAs (i.e., non-functioning, hormone-secreting, invasive, and non-invasive), we summarize the altered expression of miRNAs, their target genes, and biological significances. Finally, we also discuss the potential role of c-miRNAs as innovative non-invasive diagnostic and prognostic biomarkers of PAs.

## 2. miRNAs in Non-Functioning PAs

NFPAs constitute approximately 30% of the PAs and are hormonally inactive tumors [36]. They are typically benign with a low growth rate, but, unfortunately, aggressive NFPAs able to invade cavernous, sphenoid sinus, or dura mater, have been also described [37,38]. Although surgery is the preferred treatment for these tumors, many patients with invasive NFPAs relapse postoperatively, even after complete tumor surgical resection [39].

Boresowicz et al. [40] investigated whether a miRNA signature could discriminate NFPA patients with invasive and noninvasive gonadotropic pituitary neuroendocrine tumors (PitNETs), one of the most frequent histopathological subtypes of NFPAs. Comparing miRNome by using next generation sequencing (NGS) and by applying receiver operating characteristics (ROC) curve analysis for the 29 identified differentially expressed miRNAs, the authors found six miRNAs (miR-184, miR-181a-2-3p, miR-93-3p, miR-574-5p, miR-185-5p, and miR-3200-5p) that could be potential biomarkers of invasiveness. Significant differences were observed only for the miR-185-5p in the validation step by quantitative real-time polymerase chain reaction (qPCR)-based assay. However, results obtained from specificity and sensitivity analysis did not confirm the usefulness of this miRNA in discriminating invasive from non-invasive NFPAs. Therefore, they concluded that no miRNA signature could serve to determine the invasive growth of these tumors.

In previous studies [41,42], the expression of the *Wnt inhibitory factor 1* (*WIF1*) gene, which encodes an inhibitor of the Wnt signaling pathway, has been found to be significantly reduced in pituitary tumors compared to normal pituitary gland specimens due to the hypermethylation of its promoter. Song et al. [43] further demonstrated that a significant downregulation of WIF1 observed in invasive NFPAs was negatively correlated with clinicopathological parameters of these tumors. Additionally, among the eleven putative miRNAs supposed to bind 3′UTR of the *WIF1* mRNA based on TargetScan and published papers on PubMed, the expression levels of miR-137 were significantly downregulated in invasive groups, and its overexpression in GH3 cells transfected with pEGFP-N1-miRNA-137 reduces both cell proliferation and WIF1 expression at both mRNA and protein levels. Altogether, these results indicate that miR-137 could play a critical role in the regulation of the Wnt signaling pathway, potentially by affecting promoter methylation status of WIF1.

In 2008, Barbieri et al. [44] reported that the chemokine (C-X-C motif) ligand 12 (CXCL12) and its receptor C-X-C chemokine receptor type (CXCR4) are overexpressed in adenomatous tissues from human pituitary, including 37 NFPAs, and that CXCL12 could promote pituitary cell proliferation. Cai et al. [45] investigated the effect of CXCL12 on miRNA expression located within the *DLK1*-*maternally expressed gene 3* (*MEG3*) imprinted locus, which have been found to be selectively downregulated in NFPAs. miR-370-3p has been shown to be downregulated in high grade NFPAs and its expression is reduced in CXCL12-stimulated cell lines by increasing the expression of high mobility group protein HMGA2, a predicted and verified target of this miRNA. Overall, their results indicate that the CXCL12/miR-370–3p/HMGA2 axis is involved in pituitary adenoma cell growth and invasiveness.

Du et al. [46] showed that the expression of miR-145-5p is significantly reduced in NFPAs and correlates negatively with the proliferation and invasiveness of NFPAs both in vitro and in vivo. Functionally, miR-145-5p could perform its antitumor function by binding to the 3′UTR of *translationally controlled tumor protein* (*TPT1*) mRNA. In addition, they demonstrated a novel molecular mechanism according to which the circular RNA cirOMA1 promotes NFPA progression by acting as a molecular sponge for this miRNA, subsequently upregulating the expression of the downstream TPT1.

Table 1 provides an overview of the deregulated miRNAs and their validated targets in NFPAs.

## 3. miRNAs in Secreting PAs

### 3.1. miRNAs in Gonadotrophinomas

By analyzing microarray data downloaded from the Gene Expression Omnibus (GEO) database containing 16 multiple endocrine neoplasia-associated rats with pituitary homozygous mutations (p27Kip1/Csknb1) and 5 normal pituitary specimens from healthy rats, Hou et al. [47] found 187 upregulated and 370 downregulated genes between these two groups, principally enriched in the “cell cycle’ and in “neuroactive ligand-receptor interaction”. Furthermore, it has been suggested that three miRNAs (miR-145, miR-53, and miR-374) could contribute to pituitary gonadotroph adenoma development by modulating the expression of these differentially expressed genes (DEGs).

Recently, D’angelo et al. [48] reported that the lncRNA ribosomal protein SA pseudogene 52 (RPSAP52) is strongly overexpressed in PAs and enhances cell proliferation, increasing the protein levels of HMGA1 and HMGA2 by acting as a ceRNA of miR-15a, miR-15b, and miR-16. One year later, the same research group [49] clarified this mechanism, demonstrating that RPSAP52 interacts with human antigen R (HuR) resulting in delocalization of the above-mentioned miRNAs from HMGA proteins toward the cyclin-dependent kinase inhibitor p21Waf1/CIP1. Additional studies have shown that this lncRNA negatively regulates *growth arrest-specific protein 8* (*GAS8*) gene, thus demonstrating its important role in contributing to pituitary tumorigenesis.

Table 2 provides an overview of deregulated miRNAs and their validated targets in gonadotropin (GN)-secreting PAs.

### 3.2. miRNAs in Prolactinomas

Based on preliminary data reporting that miR-7a2 deficiency is responsible for reduced prolactin expression, LaPierre et al. [50] attempted to investigate the role of this miRNA in lactotrophic cell development and prolactin hormone production. They found, unexpectedly, that miR-7a2 knockout caused an early increase in lactotroph cell proliferation and prolactin production during embryonic development, followed by a subsequent reduction in the hormone’s production in adulthood. In contrast, overexpression of miR-7a2 caused delayed lactotroph development. Further studies, conducted in different mouse pituitary cell types and rat prolactinoma cells, showed that miR-7a2 acts through the repression of its target gene Raf1, a positive regulator of prolactin production, underscoring the complexity of the regulation of production of this hormone. However, future studies will need to further elucidate the role of miR-7a2 in the pathological condition of hyperprolactinemia and to determine whether it could be used as a possible therapeutic target.

Five independent studies have been performed to discover the involvement of miRNAs in prolactinoma drug resistance.

Wu et al. [51] studied the involvement of miR-93 in resistant prolactinomas to dopamine agonists (DAs), such as cabergoline (CAB). They found that, in MMQ and GH3 cells, this miRNA was able to target autophagy-related gene 7 (ATG7) protein, leading to cell autophagy inhibition and CAB resistance, while the downregulation of miR-93 had the opposite effects, with subsequent increase in the apoptosis process.

Two years later, the same research group [52] discovered that long non-coding RNA (lncRNA) H19 acts synergically with DA on prolactinomas by sponging the miR-93 and increasing the expression of ATG7 and apoptotic process. These results identified the involvement of the H19/miR-93/ATG7 axis in prolactinoma-DA resistance, which could be used as a possible future therapeutic target.

Another study conducted by Hu et al. [53] showed that miR-93-5p was upregulated in prolactinomas with a high degree of fibrosis, a process involved in drug-resistance, compared to samples without fibrosis. Subsequent research discovered that miR-93-5p mediates the onset of fibrosis through the inhibition of the *Smad7* gene and the increase of TGF-β1/Smad3 signaling.

Xiao et al. [54] investigated the mechanism of miR-1299 in drug-resistant prolactinomas. They found that this miRNA targeted the Forkhead box protein O1 (FOXO1), which binds to the prolactin gene promoter, thus inhibiting its expression. Moreover, in vitro experiments carried out on miR-1299 overexpressing primary tumor cells confirmed that prolactinomas resistant to drugs presented both an increase of PRL secretion and FOXO1 reduction.

The purpose of a study by Jian et al. [55] was to elucidate the relationship between miR-145-5p low expression, TPT1 high expression, and the resistance of prolactinomas to treatment with the dopamine agonist bromocriptine (BRC). They demonstrated that miR-145-5p directly targets TPT1, which is upregulated in BRC-resistant-prolactinoma cells. Furthermore, the downregulation of miR-145 and TPP1 upregulation are responsible for decreased BRC sensitivity both in MMQ cells and in vivo.

The findings obtained by these five studies support the theory that miR-93, miR-1299, and miR-145 may be considered potential targets to reduce drug resistance in prolactinomas.

The aim of a study by Zhang et al. [56] is to understand the function of miR-130a-3p in PRL regulation. They showed that GH3 cells transfected with the miR-130a-3p mimic had an increase of PRL expression. In addition, authors investigated its mechanism of action, discovering that miR-130a-3p represses estrogen receptor α (Erα) by targeting its 3′UTR and leading to PRL reduction. Moreover, this study highlighted that heat stress increased miR-130a-3p levels while it reduced PRL and ERα expression, both in vitro and in vivo. This evidence provides a new mechanism by which miRNAs could act in PRL regulation.

Lei et al. [57] found that miR-137 acts as a tumor suppressor in prolactinoma cells. In fact, this miRNA can inhibit MMQ and GH3 cell survival, proliferation, and migration, and reduces the volume of tumor in F344 rat prolactinomas. Bioinformatic analysis and subsequent luciferase assay confirmed that miR-137 binds and inhibits microphthalmia-associated transcription factor (MITF), which promotes cell proliferation, migration, and survival, both in vitro and in vivo. Furthermore, it was seen that miR-137 could promote Wnt-inhibitory factor-1 upregulation, suppressing the β-catenin nuclear translocation and, consequently, cell proliferation. In conclusion, all these results showed that miR-137 could play an important role as tumor suppressor in prolactinomas tumorigenesis and invasiveness, representing a future potential therapeutic target for treating this disease.

Table 3 provides an overview of deregulated miRNAs and their validated targets in PRL-secreting PAs.

### 3.3. miRNAs in Somatotropinomas

Acromegaly is a pathological condition often caused by the presence of growth hormone-secreting PAs (GHPAs). Although studies reported that this disease is partially due to an excessive function of the GH and IGF1 hormones, the pathogenesis is still not entirely clear. In their study, Xiong et al. [58] demonstrated that GHPA-derived exosomes could be involved in bone formation both in vitro and in vivo, due to their ability to increase osteoblast proliferation and viability. Furthermore, the authors observed that exosome-derived miR-21-5p is able to stimulate osteoblastogenesis in a GH/IGF1 axis-independent manner, probably by targeting the SMAD7 signaling pathway. In conclusion, these results provide a novel mechanism of action by which GHPA influences bone metabolism in patients with acromegaly and suggest exosomal miR-21-5p as a candidate target for the treatment of this disease.

Recent studies have reported that GHPAs with a mutation in the aryl hydrocarbon *receptor-interacting protein (AIP)* gene are more aggressive and resistant to treatment with drugs, such as the somatostatin analogue, octreotide [59]. Based on this evidence, Bogner et al. [60] investigated the possible involvement of miRNAs in the proliferation and drug resistance of somatotropinoma patients with an *AIP* gene mutation. The array analysis showed that the expression of miR-34a and miR-145 was upregulated in samples of patients carrying the mutation compared to control samples. Subsequently, the authors found that GH3 cells overexpressing miR-34a showed an increase in cell proliferation and production of intracellular cAMP, a molecule crucial for the process of mitosis in PAs, and a reduction of cell apoptosis. Instead, these processes were moderately affected by miR-145. Subsequent studies showed that miR-34a was able to target the mRNA of the *Gnai2* gene, which encodes for the Gαi2 subunit of the G protein, an inhibitor of cAMP production, which was found to be downregulated in GHPA samples positive for *AIP* gene mutation, as opposed to those of cAMP, which were elevated. Furthermore, cells with increased miR-34a expression showed resistance to octreotide treatment. Therefore, the results obtained in this study demonstrated that miR-34a could be considered a possible therapeutic target for the treatment of GHPA patients carrying mutations in the *AIP* gene.

Considering the increased risk of bone fragility and vertebral fractures in patients affected by acromegaly, Belaya et al. [61] studied the effects of the constant excess release of GH and insulin-like growth factor-1 (IGF1) on gene and miRNA expression in bone tissue samples of patients suffering from active acromegaly. The data obtained from this work highlighted that there are no significant differences in expression of key genes involved in osteoblast and osteoclast function, except for alkaline phosphatase (ALP) and TWIST1 mRNAs, which were downregulated by approximately 50% in bone tissue of acromegalic patients. Furthermore, it was seen that the excess of GH leads to a substantial augmentation of WNT10B and Dickkopf 1 (DKK1) expression, agonist and antagonist of the Wnt signaling pathway, respectively. Moreover, bone samples derived from acromegalic patients presented changes in the expression of miRNAs related to the commitment of mesenchymal stem cells (miR-199a-5p, miR-27-5p, miR-125b-5p, miR-34a-5p, miR-188-3p, miR-210-5p, miR-135a-5p, miR-211, miR-23a-3p, and miR-204-5p), which was shifted toward the formation of chondrocytes or adipocytes, at the expense of osteoblast differentiation. All these findings revealed that acromegaly is a negative condition for bone tissue, which suffers from progressive deterioration in quality, leading to more fragile bones.

Armagan et al. [62] investigated the genotype distribution of single nucleotide polymorphisms (SNPs) in miR-23b and miR-107, two miRNAs regulating the *HMGA2* and *AIP* gene expression, respectively, whose expression levels correlated either with GHPAs and response to treatment with somatostatin analogs (SSAs), or SNPs in 3′-UTR of HMGA2 gene in patients with acromegaly. In particular, they did not find polymorphisms in the rs201749160 region of the *HMGA2* gene in their study group, while just one healthy patient presented SNPs in the rs11185776 region of *AIP* gene. Furthermore, authors found that no relationship existed between the polymorphisms in the rs1351394 region in the miRNA binding site of HMGA2 3′-UTR and the resistance of GHPAs to treatment with SSAs in patients with acromegaly. On the contrary, polymorphic T allele was associated with higher expression of IGF-1, p53, and Ki-67, which could be considered valid biomarkers for acromegaly.

DeVore et al. [63] found high levels of both peptidyl arginine deiminase (PAD) 2 and PAD4, which are enzymes converting histone arginine residues into citrulline, thus regulating chromatin structure and gene expression, and citrullinated histones in human prolactinomas and somatoprolactinomas. In addition, this study reveals that histone citrullination induced by PADs downregulate the expression profiles of three tumor suppressor miRNAs (let-7c-2, miR-23b, and miR-29c), which are predicted to directly target oncogenes involved in somatoprolactinomas and prolactinomas pathogenesis, such as *insulin-like growth factor 1 (IGF-1)*, *HMGA1*, and *N-MYC*.

Yang et al. [64] showed in their study that the expression levels of miR-34a were significantly lower in rat pituitary adenoma cell line GH4C1 than in normal pituitary *Rattus norvegicus*-derived tissues, and its overexpression inhibited cell proliferation and promoted apoptosis by suppressing SRY-Box Transcription Factor 7 (SOX7).

Table 4 provides an overview of deregulated miRNAs and their validated targets in GH-secreting PAs.

### 3.4. miRNAs in Corticotropinomas

Cushing’s syndrome (CS) is a potentially fatal disease often caused by the presence of ACTH-secreting PAs, which leads to the onset of Cushing’s disease (CD), or, more rarely, by the existence of either primary adrenal gland disease or ACTH-producing carcinoid tumors, which determine ectopic ACTH secretion (EAS) [65].

In their study, Ren et al. [66] found that secreted angioinhibitory factor thrombospondin-1 (TSP-1), a glycoprotein responsible for mediating cell-to-matrix and cell-to-cell interactions, was downregulated in human pituitary corticotropinomas compared to normal tissue. Subsequently, they demonstrated that TPS-1 overexpression in the murine AtT20 corticotroph tumor cell line resulted in a decrease both of corticotroph precursor hormone proopiomelanocortin (POMC) synthesis and secretion of adrenocorticotropic hormone (ACTH), and suppressed pituitary adenoma cell proliferation, migration, and invasion. Further studies highlighted that miR-449c inhibits *TSP-1* by directly targeting its mRNA, thus promoting pituitary tumorigenesis. In addition, they discovered lower levels of lncRNA lncTHBS1 in patients with CS, which has been subsequently shown to be a potential negative regulator of miR-449c. Taken together, these findings suggest that lncTHBS1 could act as a competing endogenous RNA (ceRNA) for miR-449c, which may represent a future target for the treatment of CD.

Table 5 provides an overview of the deregulated miRNAs and their validated targets in ACTH-secreting PAs.

## 4. miRNAs in Invasive PAs (IPAs) and Non-Invasive PAs (NIPAs)

Some PAs are known to invade and infiltrate surrounding tissues, such as meninges, cavernous sinuses, brain, and bone tissue. Since few studies have been conducted on the molecular mechanisms and prognosis of bone-invasive PAs (BIPAs), Zhu et al. [67] tried to explore these fields. A first clinical analysis of 274 follow-up patients with different PAs revealed that bone invasion was mainly correlated with female sex, non-gross total resection (NGTR), large tumor volume, and tumor regrowth. In addition, worse progression-free survival (PFS) was observed in patients with BIPAs in the NGTR and NFPA groups compared to those with non-bone-invasive PAs (NBIPAs). Subsequently, authors discovered, by using transcriptome microarray analysis, that several mRNAs, lncRNAs, miRNAs, and circRNAs were differentially expressed between BIPA and NBIPA groups. Furthermore, the results obtained by GO and KEGG analysis revealed that immune and inflammatory pathways were the biological processes primarily involved in BIPA pathogenesis. In particular, further investigation showed that lncRNA SNHG24, NR_033258, miR-181c-5p, and miR-454-3p could regulate the expression of tumor necrosis factor α (TNFα) in BIPAs, a crucial molecule of osteoclast differentiation and immune and inflammatory responses. Therefore, authors demonstrated that these biological mechanisms could play an important role in BIPAs, providing new potential targets (i.e., TNFα and its related ncRNAs) for future treatment of neoplasms.

The aim of a study by Shen et al. [68] was to investigate the function of miR-543 in PA tumorigenesis. Their results showed that PA tissues derived from a total of 137 patients exhibited increased levels of miR-543 and decreased levels of Smad7. Bioinformatic analysis and luciferase assay confirmed that *Smad7* mRNA was a direct target of miR-543. Moreover, ectopic overexpression of miR-543 in HP75 cells enhanced cell proliferation, migration, and invasion, while reducing the apoptosis process together with cleaved caspase-8 and cleaved caspase-3 expression levels. Opposite data were observed with miR-543 downregulation. Taken together, these findings suggest that miR-543 acts as a tumor suppressor in PAs by negatively regulating Smad7 and, consequently, the Wnt/β-catenin signaling pathway.

The aim of a study by Zhang et al. [69] was to investigate the involvement of miRNAs in invasion of PAs. The differential analysis of miRNA expression among IPA and NIPA samples revealed that 31 miRNAs were upregulated while 24 were downregulated in invasive groups compared to non-invasive ones. Function enrichment studies, using programs such as GO and KEGG, showed that these miRNAs target genes associated mainly with cell cycle, proliferation, and apoptotic process. In addition, the miRNA-mRNA network, generated by Cytoscape software, highlighted 258 predicted target genes, 7 downregulated miRNAs, and 5 upregulated miRNAs in IPAs, including miR-665 and miR-149-3p, which exhibited an opposite trend but, interestingly, were involved in the same biological processes, such as cell proliferation and invasion. Although further studies will have to be performed to confirm these bioinformatic predictions, this study provided new insights regarding the role of miRNAs in IPA pathogenesis, which could be considered new possible future targets for the treatment of these tumors.

According to a report by Grzywa et al. [70], miR-410-3p acts as an oncomir in corticotroph and gonadotroph PAs, while it exhibits oncosuppressor properties in somatotroph pituitary adenoma. In particular, this miRNA promotes proliferation and invasiveness in gonadotroph and corticotroph adenoma cells through regulating phosphatase and tensin homologue (PTEN)/AKT, mitogen-activated protein kinase (MAPK), and activator of transcription 3 (STAT3) signaling pathways, while it has an opposite effect on somatotroph pituitary adenoma cells. Moreover, the levels of miR-410-3p are found to be the highest in somatotroph PAs compared to those of gonadotroph, while the lowest are observed in gonadotroph and null cell adenomas. These results suggest that miR-410-3p may act differentially either as a tumor suppressor or an oncomiR, depending on the biological context.

Given the implication of caveolin-1 (Cav-1) in cancer progression, Yang et al. [71] studied its possible involvement in pituitary adenoma invasion mechanism. They found that the Cav-1 knockout (KO) cells lost their ability to invade and showed a gain in expression of miR-124, miR-145, and miR-183. Subsequent studies revealed that Cav-1 KO inhibited EGR1 nuclear translocation and its interaction with KLF5 factor, which remains free and is able to promote the expression of the above-mentioned miRNAs. Moreover, the authors discovered that miR-124, miR-145, and miR-183 target *PTTG1IP*, *FSCN1*, and *EZR* genes, respectively, whose suppression led to the inhibition of pituitary adenoma cell migration and invasion. In conclusion, this study demonstrated that Cav-1 promotes PA invasiveness by regulating the functions of EGR1 and KLF5 factors, and may be a promising target for the development of new therapeutic strategies against IPAs.

Wang et al. [72] found that invasive pituitary tumor cell lines and tissues are characterized by lnc-SNHG1 overexpression, which promoted cell proliferation, migration, invasion, and epithelial-mesenchymal transition (EMT) in vitro and tumor growth in vivo. Subsequent studies evidenced that lnc-SNHG1 was able to sponge a miRNA-pool composed of miR- 302, miR-372, miR-373, and miR-520, thus inhibiting their activity and enhancing the expression of their gene targets, *TGFBR2* and *RAB11A*, which promote TGF/SMAD3 and Wnt/β-catenin signaling pathways, respectively. All these findings demonstrated that lnc-SNHG1 is involved in the carcinogenesis of IPAs via sponging miR-302/miR-372/miR-373/miR-520, helping to better understand the molecular mechanisms of the development and aggression of IPAs and providing new possible molecules with diagnostic and therapeutic value.

One year later, Zhu et al. [73] identified lncRNA MEG3 as a possible factor involved in pituitary tumor invasiveness. In fact, their study highlighted that expression levels of this lncRNA and its downstream miRNA, miR-376B-3p, were diminished in patients with clinical NFPAs (CNFPAs) compared to normal pituitary samples. In addition, it was observed that the transcriptional levels of MEG3 and miR-376B-3p were significantly lower in the non-invasive CNFPAs compared to the invasive group. Moreover, the authors discovered that MEG3 overexpression in the pituitary cell line PDFS leads to tumorigenesis inhibition and a substantial increase in apoptosis rate. Subsequent findings revealed that MEG3 could induce miR-376B-3P transcription, which could act as a tumor suppressor by binding and inhibiting its target oncogene, *HMGA2*. In summary, this study affirmed that the MEG3/miR-376B-3p/HMGA2 axis could be involved in CNFPA phenotypic regulation and potentially help the development of new therapeutic strategies against PAs. 

Zhao et al. [74] explored the role of miRNAs in IPAs and the possible effectiveness of miRNA-exosome strategy for treating this disease. Among the 20 differentially expressed miRNAs between IPAs and NIPAs, miR-99a-3p and miR-149-5p were the lowest expressed in IPAs, so these were selected for the subsequent studies. They discovered that the expression levels of MMP-2, MMP-9, Vimentin, N-cadherin, and VEGF were upregulated, while those of p53 and E-cadherin were downregulated in IPAs. Further research evidenced that the downregulation of miR-99a-3p and miR-149-5p increased the viability, migration, and invasion of GH3 and MMQ cells, while their overexpression led to opposite results. In addition, authors found that HUVEC cells, co-cultured with MMQ or GH3 cells treated with miR-19-5p and miR99a-3p inhibitors, respectively, showed increased cell ability to produce longer tubes, as opposed to what was observed in the co-culture of HUVECs with MMQ and GH3 cells transfected with miRNAs mimics. The effects of exosome-derived miR-99a-3p and miR-149 overexpression were investigated in MMQ and GH3 on pituitary tumors, discovering that both miR-99a-3p and miR-149 were able to suppress cell viability, migration, and tube formation in vitro. Their effects were also studied in vivo, where a decrease in tumor growth and expression levels of angiogenesis-related marker was observed. Finally, they discovered that miR-99a-3p target *NOVA1*, *DTL*, and *RAB27B* genes. In conclusion, this study identified miR-99a-3p and miR-149 as suppressors of IPA tumorigenesis and demonstrated that treatment with exosome-miRNAs could represent a valuable potential therapy for IPA medical care.

Duan et al. [75] investigated the role of miR-137 in PAs. They found that its expression levels were significantly downregulated in pituitary tumor tissues versus normal pituitary tissues, and particularly in those which show invasive capacity. Cells transfected with miR-137 mimics exhibited both a lower invasive capacity and a reduced growth rate compared with negative control, while its suppression reversed these effects. Functionally, miR-137 exerts its role as a tumor suppressor miRNA through a negative regulation of AKT2 expression.

A report from Zhao et al. [76] recently found that expression levels of LncRNA PCAT6 and BRD4 were increased, while those of miR-139-3p were lower in IPA tissues. In vitro experiments revealed that miR-139-3p mimic, siBRD4, and shPCAT6 inhibited the proliferation, viability, migration, and invasion, while they promoted cell apoptosis in pituitary adenoma cells RC-4B/C and GH3. Moreover, administration of miR-139-3p mimic and shPCAT6 in nude mice inhibited the increase of tumor volume and weight, prevented cell cycle progression, and promoted cell apoptosis by upregulating the expression of miR-139-3p, Bax, and cleaved-caspase 3 and by downregulating that of BRD4 and Bcl-2. These effects were reversed through the introduction of miR-139-3p inhibitor. These findings suggest that lncRNA PCAT6 could play a crucial role in PA tumorigenesis via targeting miR-139-3p/BRD4 axis.

Even the lncRNA X-inactive specific transcript (XIST) has been reported to promote the development of PitNET by functioning as a ceRNA of miR-424-5p. Zhou et al. [77] observed that the expression levels of XIST were higher in invasive tissues compared with non-invasive PitNETs along with that of basic fibroblast growth factor (bFGF), while those of miR-424-5p were significantly lower. Additionally, XIST promotes the proliferation, migration, and invasion, and suppresses cell cycle arrest and apoptosis, of invasive PitNET cells by competitively binding to miR-424-5p, thereby leading to an increase in bFGF levels. In conclusion, these molecules could be possible innovative therapeutic targets for invasive PitNET.

Table 6 provides an overview of deregulated miRNAs and their validated targets in IPAs.

## 5. miRNAs Deregulated in a Variety of Pituitary Tumors

### 5.1. miRNA Expression Profiling in Human Samples from Patients with Pituitary Tumors

Several genes involved in aggressive PRL pituitary tumors have been shown to be under the transcriptional regulation of E2 transcription factor 1 (E2F1), which in turn is regulated by MYC and some miRNAs [15,78,79]. Therefore, García-Martínez et al. [80] explored the relationship between the expression of E2F1 and miR-17-92 cluster (miR-17-5p and miR-20a) with the proliferation and invasiveness of different subtypes of pituitary tumors. Their results show that E2F1 expression is increased in gonadotroph PAs (GT) and silent corticotroph PAs (sCT), while it is expressed normally in functioning CT and somatotroph PAs (ST). Moreover, its expression is higher in invasive tumors compared to non-invasive ones in ST and in the whole series. MYC expression is higher in invasive compared to non-invasive ST, while it is lower in invasive GT with respect to non-invasive ones. miR-17-5p expression levels have been shown to be more expressed in proliferative tumors when compared to non-proliferative ones in the whole series, but not according to the subtypes. In conclusion, they suggest miR-17-5p and E2F1 as promising biomarkers of proliferation and invasiveness of pituitary tumors, respectively, thus helping the clinical management of PitNETs.

RNA sequencing analysis of samples obtained from PRL- and GH-secreting PA patients and NFPA patients showed differentially expressed miRNA profile compared to those of normal pituitary subjects. Following qPCR assay-based validation, He et al. [81] detected a specific miRNA signature concerning various subtypes of PAs. In NFPA samples, the levels of miR-493-5p were found lower, while those of miR-181b-5p were significantly increased. In GH-secreting samples, an increase in miR-184 expression was noticed. In PRL-secreting samples, miR-34c-3p expression was significantly decreased, together with that of miR-34b-5p, miR-378, and miR-338-5p. In addition, they found a low expression of miR-124-3p in both GH-secreting and NFPAs. Taken together, these distinctive patterns of miRNA expression could be used as novel potential therapeutic biomarkers for each pituitary adenoma subtype.

Given the importance of glucose-6-phosphate dehydrogenase (G6PD) in cancers, the rate-limiting enzyme of the pentose phosphate pathway (PPP), He et al. [82] investigated the involvement of miR-1, previously found to negatively regulate G6PD [83], in pituitary tumor. The levels of miR-1 have been shown to be reduced in pituitary tumor cells and tissues compared to control group, and its lower expression is significantly associated with poor prognosis and tumor aggressiveness. miR-1 overexpression in HP75 and MMQ cells transfected with miR-1 mimics represses cell proliferation by targeting G6PD to suppress the PPP and inhibit the glucose metabolism of cancer cells. Their results show that either miR-1 or G6PD may serve as potential therapeutic targets against human pituitary cancer.

### 5.2. miRNA Expression Profiling in Mouse (AtT-20 and GT1.1) and Rat (GH3 and MMQ) Pituitary Adenoma Cell Lines

Based on previous studies, miR-205-5p has been indicated to play an antitumor role in different types of cancers, including prostatic carcinoma, prostate cancer, and oral squamous cell carcinoma [84,85,86]. Hu et al. [87] demonstrated that its expression was downregulated in pituitary adenoma cell lines GH3 and MMQ with respect to a normal cell line. In addition, miR-205-5p overexpression inhibits cell proliferation and migration by negatively regulating the *chromobox homolog 1* (*CBX1*) mRNA, suggesting this miRNA as a potential therapeutic target for this pathological condition.

Tang et al. [88] found that silencing of the lncRNA actin filament-associated protein 1-antisense RNA 1 (AFAP1-AS1) represses cell proliferation and promotes apoptosis of pituitary tumor cells through regulating the PI3K/AKT signaling pathway. One year later, the same research group [89] demonstrated that this lncRNA affects the proliferation by acting as a ceRNA of miR-103a-3p thus regulating the PI3K/AKT signaling pathway. Meanwhile, in vitro experiments on cells co-transfected with rno-miR-103a-3p inhibitor and si-AFAP1-AS1 revealed an increase of both cell cycle progression and proliferation, a reduced cell apoptosis, an increase of PRL and GH secretion, as well as an upregulation of PI3K/AKT signaling transduction pathway in pituitary adenoma rat-derived cell lines.

Wang et al. [90] showed that the levels of miR-219a-2-3p were significantly downregulated in mouse pituitary adenoma cells AtT-20 and GT1.1 in comparison with pituitary cell MPC. Moreover, miR-219a-2-3p overexpression by transfecting with miRNA mimics into both pituitary adenoma cell lines resulted in suppressed cell proliferation and enhanced cell apoptosis by the regulation of mouse double minute 2 (MDM2)/p53 axis. These findings suggest that miR-219a-2-3p could serve as novel biomarker and treatment of PA patients.

### 5.3. miRNA Expression Profiling in MEN1-Associated Pituitary Adenomas

Multiple endocrine neoplasia type 1 (MEN1) is a rare inherited syndrome characterized by the occurrence of pituitary, parathyroid, and pancreatic tumors in a single patient [91]. Regarding MEN1-associated PAs, Lines KE et al. [92] studied the levels of three miRNAs, miR-16-1, miR-15a, and let-7a, demonstrated to be downregulated in different subtypes of non-MEN1 PAs [16,93,94,95,96,97], in pituitary neoplasms from heterozygous *MEN1* mutant mice (*MEN1^+/−^* mice). All three miRNAs were downregulated in *MEN1^+/−^* mice compared with normal wild type pituitaries. In addition, the expression of miR-16-1 and miR-15a inversely correlated with that of cyclin D1 (CCD1), thus proving previous reports that indicated this protein as a possible target of both these miRNAs [98,99,100]. They further demonstrated that menin-knockout HeLa and AtT20 cells have significantly reduced miR-15a levels. In conclusion, miR-15a and miR-16-1 represent promising molecular targets to develop a novel approach to blocking pituitary tumorigenesis in MEN1 patients.

Consistent with these findings, the exploration of miRNAs and the underlying mechanisms related to pituitary tumorigenesis might lead to the identification of novel potential biomarkers, which could be used as innovative therapeutic options to treat pituitary tumors.

Table 7 provides an overview of deregulated miRNAs in a variety of pituitary tumors.

## 6. Circulating miRNAs in PAs

Based on previous studies, in which differential expression of miR-145, miR-21, miR-141, let-7a, miR-150, miR-15a, miR-16, and miR-143 was observed in ACTH-secreting pituitary tumors compared to healthy pituitary tissues [94], Belaya et al. [101] assessed their expression profile in the plasma of patients affected by ACTH-dependent CS caused by either EAS or CD. They found altered levels of miR-16-5p, mir-145-5p, and miR-7g-5p, which are more expressed in plasma of patients with CD compared to patients with EAS. In addition, plasma levels of miR-16-5p exhibited the most differentiation power between patients with CD and EAS with an AUC value of 0.879 in ROC curve analysis. In conclusion, these data provide possible c-miRNAs that could be used as potential biomarkers to distinguish CD and EAS patients.

A study by Németh et al. [102] found fifteen differentially expressed plasma miRNAs according to adenoma subtypes, while the expression of fourteen miRNAs was altered in the plasma of patients with PAs compared to healthy controls. By comparison of miRNA expression in plasma samples obtained from pre-operative and late post-operative clustered into different PA subtypes by using NGS analysis, the authors identified, respectively, three, seven, and sixty-six differentially expressed miRNAs in GH-secreting, GT, and hormone-immunonegative groups. However, only miR-143-3p was proven to be expressed significantly lower in the late post-operative group compared to the preoperative one, but exclusively in the FSH/LH^+^ group in the qPCR-based validation phase. ROC curve analysis confirmed its diagnostic potential in discriminating pre- and post-operative patients with an area under the curve (AUC) value of 0.79. In conclusion, a global downregulation of plasma miRNAs levels is observed between pituitary adenoma patients and healthy controls, and, in particular, miR-143-3p could be a promising biomarker for evaluating patient follow-up only for the FSH/LH^+^ group, although further investigations are required to confirm these data.

The expression of matrix metalloproteinase-9 (MMP9) and vascular endothelial growth factor (VEGF) have been suggested to be closely related to pituitary tumor growth, invasion, and metastasis [103,104]. Lu et al. [105] reported decreased expression of miR-16 in serum from the patients affected by pituitary tumors compared with healthy group, and longer overall survival (OS) and disease-free survival (DFS values) are observed in patients with higher levels of this miRNA. Furthermore, they found that elevated expression of miR-16 enhances cell apoptosis and suppresses both cell proliferation and angiogenesis by regulating the expression of p27, Bax, VEGF receptor-2 (VEGFR-2)/p38/NF-κB signaling pathway, and the activities of caspase-3/8 in human pituitary cancer HP75 cells transiently transfected with miR-16 mimics. Therefore, miR-16 could be a potential therapeutic target for pituitary cancer.

Zhao et al. [106] tried to identify new c-miRNAs that could be used as biomarkers for the diagnosis, prognosis, and therapy of somatotropic adenomas. They found that 169 serum exosomal miRNAs were differentially expressed in somatotroph adenoma samples compared to normal controls. In particular, miR-423-5p was significantly downregulated in the first group, while the expression of Synaptotagmin 1 (SYT1) and pituitary tumor transforming gene (PTTG1) was upregulated. While SYT1 was not reported to be a target of miR-423-5p, PTTG1 was seen to be a direct target of this miRNA. Further studies revealed that overexpression of PTTG1 may contribute to the promotion of proliferation and migration of somatotropic adenoma cells. In conclusion, this study proposes that c-miRNAs, especially miR-423-5p, and PTTG1 could be considered valuable biomarkers for the development of new therapeutic strategies against somatotropic adenomas.

These studies highlight that the identification of a specific c-miRNA signature would be an important step not only for diagnosis and prognosis, but also to develop effective and personalized therapeutic strategies for patients with PAs. However, there are a few available studies concerning the use of c-miRNAs as novel potential biomarkers in PAs and their translation from basic research to clinical practice.

Table 8 provides an overview of deregulated c-miRNAs in PAs.

## 7. Discussion

PAs are one of the most common intracranial tumors [107]. They are most often benign neoplasms, although malignant forms of PAs have been documented to invade surrounding tissues [108]. Clinical manifestations often take the form of visual disturbances and systemic endocrine disorders, but these symptoms are not always present in patients, thus leading to a delay in tumor diagnosis and treatment. Typically, conventional therapeutic approaches include surgical resection of the adenoma, treatment with specific drugs or, in particular cases, treatment with radiation therapy [7]. Although these methods lead to a reduction in tumor volume and symptoms, it is not always possible to completely remove the cancer tissue, thus exposing the patient to possible recurrences. In addition, the drugs used against PAs may cause important collateral effects, such as nausea, dizziness, stomach pain, vomiting, diarrhea, gall bladder stones, headache, fatigue, and pain at the site of injection [6].

For these reasons, the scientific community is shifting its attention toward the identification of new possible biomarkers that could be used in diagnosis, prognosis, and therapy in PAs. In the past few years, it has been reported that one or more epigenetic mechanisms play a critical role in controlling pituitary cell growth and survival [109]. Epigenetics is a set of heritable molecular mechanisms involved in the regulation of gene transcription, independent of changes in the DNA nucleotide sequence. It encompasses several biological processes, such as histone modifications, DNA methylation, and gene silencing by noncoding RNAs (ncRNAs), including miRNAs [110]. The latter are small molecules of 18-25 nucleotides able to regulate gene expression at the post-transcriptional level, either by inducing degradation or by inhibiting the translation of mRNA target [17,18].

In 2005, Bottoni et al. [16] were the first to show that there is a differential expression of several miRNAs between normal pituitary tissues and PAs. Since then, several studies have reported the possible involvement of miRNAs in PA tumorigenesis, invasion, and aggressiveness, demonstrating that some are able to behave as tumor suppressors and others as oncomiRs in PAs, depending on the biological context, thus considering them to be possible therapeutic agents [2,6]. Furthermore, some studies have shown that miRNA expression can be modulated by bromocriptine treatment in lactotroph tumors [111,112] and by hormone therapy drugs [113], thereby suggesting their involvement in mechanisms related to pharmacotherapy and their potential for the future development of miRNA-based therapeutic strategies.

Moreover, two different strategies could be adopted to restore normal miRNA levels in PAs, such as the use of miRNA inhibitors to decrease the expression levels of oncomiRs, or the use of miRNA mimics to increase the expression levels of tumor suppressor miRNAs [114]. miRNA mimics are synthetic double-stranded miRNA-like RNA designed to integrate into the RNA-induced silent complex (RISC), mimicking the function of mature endogenous miRNA. There are several molecules that have been developed in recent years regarding the suppression of oncomiR expression, such as antisense oligonucleotides (AMOs), blocked nucleic acids (nucleotide molecules containing a fragment of LNA modified to improve their specificity and stability), miRNA masks (oligonucleotides complementary to the mRNA target binding sequence and modified with 2′-OMe groups), low molecular weight miRNA inhibitors (SMIRs), and antagomiRs (oligonucleotides conjugated to a cholesterol molecule to facilitate cell uptake) [114].

Overall, we have shown that several deregulated miRNAs and their target genes have been described in PAs, but probably further genes associated with the pathogenesis of PAs are regulated by these molecules. We have additionally revealed that the aberrant expression of miRNAs is commonly associated with altered cellular functions, such as cell proliferation, apoptosis, migration, and invasion. However, the research in this field is still in its infancy and there is much that needs to be investigated before definite evidence able to prove the role of miRNAs in PAs pathogenesis.

**Figure 1:**
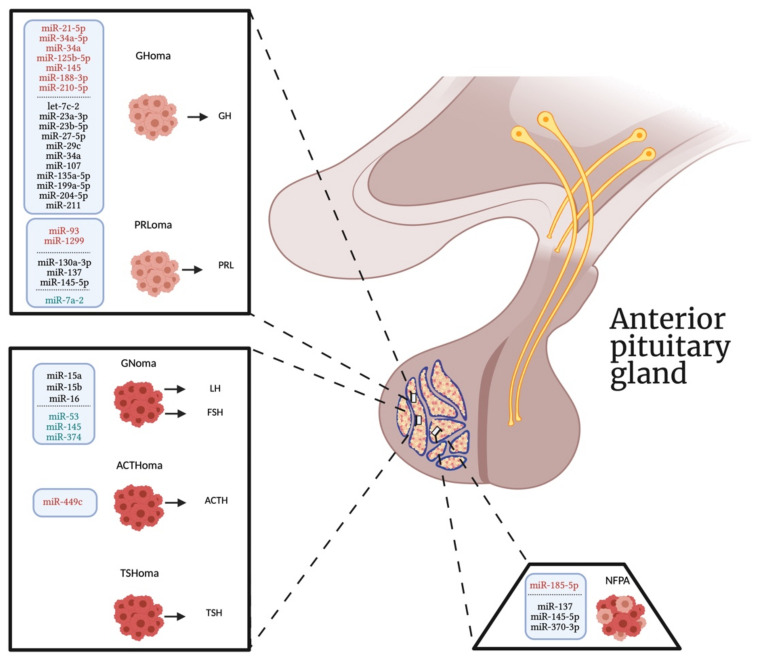
The altered expression of miRNAs according to the subtype of PAs. Red, black, and green colors indicate upregulated miRNAs, downregulated miRNAs, and dysregulated miRNAs levels, respectively. GHoma, growth hormone-secreting pituitary adenomas; PRLoma, prolactin-secreting pituitary adenomas; GNoma, gonadotropin-secreting pituitary adenomas; ACTHoma, adrenocorticotropic hormone-secreting pituitary adenomas; TSHoma, thyroid-stimulating hormone-secreting pituitary adenomas; NFPA, non-functioning pituitary adenomas.

However, it is noteworthy that none of these studies detected the same differentially expressed miRNA according to the different subtype of PAs (Figure 1), in agreement with previous findings showing that each PAs histotype showed distinct miRNA expression patterns [93]. Given that miRNAs expression seems to be cell-type specific, the comprehension of their mechanisms of actions in other cell types could be important to determine their possible off-target effects, particularly before translation the utility of miRNAs in clinical practice. Moreover, increasing findings indicate that miRNAs may be involved in the mechanism of action of some pituitary target drugs, such as the dopamine agonists and the somatostatin analogs (SSAs) [111,113]. Therefore, it is possible that the levels of specific miRNAs could be used in the future for monitoring therapeutic response to the available drugs, thus helping to design a personalized therapy for each individual patient.

Recently, in addition to intracellular miRNAs, c-miRNAs captured the attention of researchers, as they have been shown to be easily detectable potential diagnostic and prognostic biomarkers in several human diseases [22,23,24,25,35].

Despite increasing evidence showed the presence of miRNAs in different biological fluids, their origin and function is still a source of query and debate. An intriguing idea is that c-miRNAs could have a crucial role in cell-to-cell communication, where specific miRNAs could be secreted and delivered to recipient cells, probably to regulate gene expression [115,116,117]. Regarding PAs, Németh et al. [102] have assumed that the expression levels of miRNAs in the blood might reflect hormonal effects even in NFPAs which can secrete inactive, defective hormones or subunits which cannot be detected by conventional hormone assays. Another option is provided by the investigations of Michael et al. [118], who showed that variations of c-miRNAs expression levels may be attributable to the decreased retention of the miRNAs in the cells.

Few studies have been conducted to assess their expression in patients with PAs so far. However, the possible identification of specific c-miRNAs associated with different subtypes of PAs could facilitate the early diagnosis and treatment of these tumors, especially in NFPAs, which are often asymptomatic [119].

Moreover, several problems need to be solved before their translatability into clinical practice as biomarkers. c-miRNA levels have been proven to be influenced by different factors related to intra- and inter-individual variations, such as ethnicity [120] and gender [121,122], and lifestyle factors, such as smoking [123], physical activity [124], diet [125,126], and drug assumption [127]. Furthermore, regarding the measurement of miRNAs in biological fluids, differences in starting material, RNA extraction yield, and reaction efficiency must also be taken into account, as well as a valid endogenous miRNA for subsequent data normalization [128]. In fact, no universal endogenous normalizer has been identified for quantifying miRNA expression in biological fluids so far.

Therefore, a potential strategy to overcome these challenges might be to conduct a massive screening of c-miRNAs, in order to not exclude them a priori. Clearly, before entering in clinical practice, the identified molecules should be validated using a larger independent cohort to determine whether such candidate miRNAs could be potential diagnostic and prognostic biomarkers in PAs.

In conclusion, we provide an update on the different intracellular miRNAs involved in the proliferation, migration, and invasiveness of different subtypes of PAs over the last three years, which could then be considered as future targets for the development of new personalized therapeutic strategies against this disease (Figure 1). In addition, the identification of a c-miRNA signature according to the different PA subtypes might be important to use these small molecules as potential biomarkers for the early diagnosis and treatment of these tumors, especially for non-secreting tumors. Further studies will need to be conducted to increase the comprehension of the cellular and underlying molecular mechanisms by which miRNAs act in the onset and progression of PAs and to demonstrate the usefulness of c-miRNAs as biomarkers with a potential diagnostic and prognostic value for this disease.

## Figures and Tables

**Table 1:** Summary of differentially expressed miRNAs with their validated targets in non-functioning pituitary adenomas.

miRNA	Expression Levels	miRNA Profiling Platform	Biological Function	Target Genes	Reference
miR-185-5p	↑	NGS, qPCR	Status of invasive growth not confirmed by ROC analysis	/ ^1^	[40]
miR-137	↓	qPCR	Regulates Wnt signaling pathway	*WIF1*	[43]
miR-370-3p	↓	RT-PCR	Increases cell proliferation and invasiveness	*HMGA2*	[45]
miR-145-5p	↓	qPCR	Increases cell proliferation and invasiveness	*TPT1*	[46]

^1^ / Data not reported.

**Table 2:** Summary of differentially expressed miRNAs with their validated targets in gonadotrophinomas.

miRNA	Expression Levels	miRNA Profiling Platform	Biological Function	Target Genes	Reference
miR-145, miR-53, and miR-374	/ ^1^	RNA-sequencing	Contribute to tumor development	DEGs ^2^ mainly enriched in cell cycle and neuroactive ligand-receptor interaction	[47]
miR-15a, miR-15b, and miR-16	↓	qPCR	Contributes to pituitary tumorigenesis	*HMGA1* and *HMGA2*	[49]

^1^ / Data not reported; ^2^ differentially expressed genes.

**Table 3:** Summary of differentially expressed miRNAs with their validated targets in prolactinomas.

miRNA	Expression Levels	miRNA Profiling Platform	Biological Function	Target Genes	Reference
miR-7a2	/ ^1^	qPCR	Regulates prolactin production	*Raf1*	[50]
miR-93	↑	qPCR	Mediates CAB resistance	*ATG7*	[51,52]
miR-93-5p	↑	RNA-sequencing, PCR	Regulates TGF-β1/Smad3 signaling-mediated fibrosis	*Smad7*	[53]
miR-1299	↑	RNA-sequencing, qPCR	Regulates PRL gene transcription participating in the drug resistance	*FOXO1*	[54]
miR-145-5p	↓	qPCR	Regulates drug resistance	*TPT1*	[55]
miR-130a-3p	↓	Microarray, qPCR	Role in prolactin regulation	*ERα*	[56]
miR-137	↓	Microarray, qPCR	Regulates Wnt-β-catenin signaling pathway	*MITF*	[57]

^1^ / Data not reported.

**Table 4:** Summary of differentially expressed miRNAs with their validated targets in somatotropinomas.

miRNA	Expression Levels	miRNA Profiling Platform	Biological Function	Target Genes	Reference
Exosomal miR-21-5p	↑	qPCR	Regulates osteoblast proliferation, collagen I and osteocalcin synthesis, and bone formation	/ ^1^	[58]
miR-34a and miR-145	↑	Microarray, qPCR	Regulates cell proliferation and GH secretion in vitro, but also mediates resistance to the antiproliferative and hormonal properties of octreotide	*Gnai2*	[60]
miR-125b-5p, miR-34a-5p, miR-188-3p, miR-210-5p, miR-27-5p miR-135a-5p, miR-199a-5p miR-211, miR-23a-3p, and miR-204-5p	↑ ↑ ↑ ↑ ↓ ↓ ↓ ↓ ↓ ↓	qPCR	Involvement in mesenchymal stem cell commitment	/ ^1^	[61]
miR-23b and miR-107	↓	qPCR	/ ^1^	*HMGA2* and *AIP*	[62]
let-7c-2, miR-23b, and miR-29c	↓	qPCR	Promotes cell proliferation	*HMGA*, *IGF-1*, and *N-MYC*	[63]
miR-34a	↓	qPCR	Promotes cell proliferation and inhibits cell apoptosis	*SOX7*	[64]

^1^ / Data not reported.

**Table 5:** Summary of differentially expressed miRNAs with their validated targets in corticotropinomas.

miRNA	Expression Levels	miRNA Profiling Platform	Biological Function	Target Genes	Reference
miR-449c	↑	qPCR	Regulates POMC transcription, ACTH synthesis, cells proliferation, migration, and invasion	*TSP-1*	[66]

**Table 6:** Summary of differentially expressed miRNAs with their validated targets in invasive pituitary adenomas.

miRNA	Expression Levels	miRNA Profiling Platform	Biological Function	Target Genes	Reference
miR-181c-5p, and miR-454-3p	/ ^1^	Microarray, qPCR	Regulate TNFα signaling pathway	/ ¹	[67]
miR-543	↑	qPCR	Regulates cell proliferation, migration, invasion, apoptosis, and Wnt/β-catenin signaling pathway	*Smad7*	[68]
55 miRNAs	31 ↑ and 24 ↓	RNA sequencing	Regulate the invasive behavior	DEGs ^2^ mainly enriched in cell proliferation and cell cycle pathway	[69]
miR-410-3p	↑ or ↓	qPCR	Regulates MAPK, PTEN/AKT, and STAT3 signaling pathways	/ ¹	[70]
miR-145, miR-124 and miR-183	↓	Microarray, qPCR	Regulate the migration and invasion	*FSCN1*, *PTTG1IP*, and *EZR*	[71]
miR-302/372/373/520	↓	qPCR	Regulate cell proliferation, apoptosis, migration, invasion, EMT, and tumor growth	*TGFBR2* and *RAB11A*	[72]
miR-376B-3P	↓	qPCR	Regulates tumor invasiveness	*HMGA2*	[73]
Exosomal miR-99a-3p and miR-149-5p	↓	qPCR	Regulate growth and metastasis of pituitary adenoma cells	*NOVA1*, *DTL* and *RAB27B*	[74]
miR-137	↓	qPCR	Promotes cell proliferation and invasion	*AKT2*	[75]
miR-139-3p	↓	qPCR	Promotes cell viability, proliferation, migration, invasion and inhibits apoptosis	*BRD4*	[76]
miR-424-5p	↓	qPCR	Promotes cell proliferation, migration, invasion and inhibits apoptosis	*bFGF*	[77]

^1^ / Data not reported; ^2^ differentially expressed genes.

**Table 7:** Summary of differentially expressed miRNAs with their validated targets in a variety of pituitary tumors.

PitNET Types	miRNA	Expression Levels	miRNA Profiling Platform	Biological Function	Target Genes	Reference
GT ^2^, CT ^3^, sCT ^4^, ST ^5^	miR-17-5p	↑	qPCR	Involvement in tumor growth and invasiveness	/ ^1^	[80]
GH ^6^, NF ^7^, PRL ^8^	miR-184 miR-34c-3p miR-34b-5p, miR-378, miR-338-5p, miR-124-3p	↑ ↓ ↓ ↓ ↓ ↓	NGS, qPCR	/ ¹	/ ¹	[81]
/ ^1^	miR-1	↓	qPCR	Promotes cell proliferation, inhibits cell apoptosis, and glucose metabolism of cancer cells	*G6PD*	[82]
GH ^6^, PRL ^8^	miR-205-5p	↓	qPCR	Contributes cell proliferation and migration	*CBX1*	[87]
GH ^6^, PRL ^8^	miR-103a-3p	↓	qPCR	Promotes rat pituitary adenoma cell proliferation and PI3K/AKT signaling pathway, inhibits cell apoptosis and PRL and GH secretion	/ ¹	[89]
GT ^2^, ACTH ^9^	miR-219a-2-3p	↓	qPCR	Promotes cell proliferation and inhibits cell apoptosis	*MDM2*	[90]
MEN1-related ^10^	miR-15a, miR-16, and let-7a	↓	qPCR	Regulate CCND1 expression	/ ¹	[92]

^1^ / Data not reported; ^2^ gonadotroph; ^3^ functioning corticotroph; ^4^ silent corticotroph; ^5^ somatotroph; ^6^ growth-hormone secreting; ^7^ non-functioning; ^8^ prolactin-secreting; ^9^ adrenocorticotropic-secreting; ^10^ multiple-endocrine neoplasia type 1-related pituitary adenomas.

**Table 8:** Summary of differentially expressed c-miRNAs in PAs.

Biological Fluid	miRNA	Expression Levels	miRNA Profiling Platform	Potential Use as Biomarker	Reference
Plasma	miR-16-5p, mir-145-5p, and miR-7g-5p	↑	qPCR	Distinguishing between CD ^1^ and EAS ^2^ patients	[101]
Plasma	miR-143-3p	↓	NGS, qPCR	Patient FSH/LH^+^ follow up	[102]
Serum	miR-16	↓	qPCR	Correlation with longer OS ^3^ and DFS ^4^ in pituitary tumor patients	[105]
Serum	miR-423-5p	↓	Microarray, NGS, qPCR	Clinical treatment of somatotroph adenomas	[106]

^1^ Cushing’s disease; ^2^ ectopic ACTH secretion; ^3^ overall survival; ^4^ disease-free survival.

## Data Availability

Not applicable.
